# Supplementary figures and images for: Pervasive loss of regulated necrotic cell death genes in elephants, hyraxes, and sea cows (Paenungualta)
Source: bioRxiv. 2024 Apr 5:2024.04.04.588129. Preprint. [Version 1] doi: 10.1101/2024.04.04.588129 (PMC11014510; doi:10.1101/2024.04.04.588129)

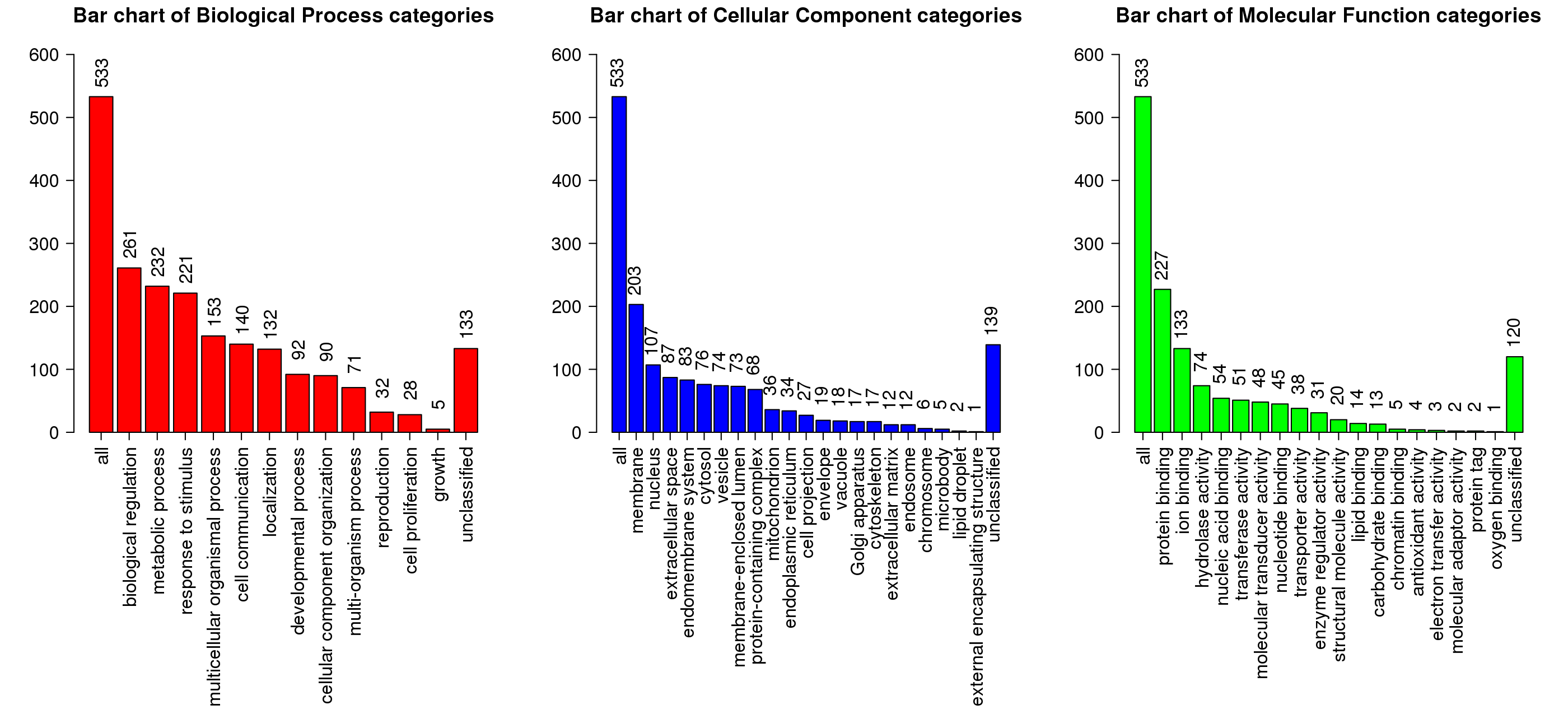

Supplement: Supplement 2 [file media-2.zip › Figure 5 ΓÇô source data 1. Reactome pathway enrichment results/goslim_summary_wg_result1709049343.png]
